# A Clinic-Radiomics Model for Predicting the Incidence of Persistent Organ Failure in Patients with Acute Necrotizing Pancreatitis

**DOI:** 10.1155/2023/2831024

**Published:** 2023-08-17

**Authors:** Nan Liu, Yidong Wan, Yifan Tong, Jie He, Shufeng Xu, Xi Hu, Chen Luo, Lei Xu, Feng Guo, Bo Shen, Hong Yu

**Affiliations:** ^1^Department of Critical Care Medicine, Sir Run Run Shaw Hospital, School of Medicine, Zhejiang University, Hangzhou, China; ^2^Center of Severe Pancreatitis, Sir Run Run Shaw Hospital, School of Medicine, Zhejiang University, Hangzhou, China; ^3^Institute of Translational Medicine, Zhejiang University, Hangzhou, China; ^4^Department of General Surgery, Sir Run Run Shaw Hospital, School of Medicine, Zhejiang University, Hangzhou, China; ^5^Department of Radiology, Sir Run Run Shaw Hospital, School of Medicine, Zhejiang University, Hangzhou, China; ^6^Department of Radiology, People's Hospital of Quzhou, Quzhou, China

## Abstract

**Background:**

Persistent organ failure (POF) is the leading cause of death in patients with acute necrotizing pancreatitis (ANP). Although several risk factors have been identified, there remains a lack of efficient instruments to accurately predict the incidence of POF in ANP.

**Methods:**

Retrospectively, the clinical and imaging data of 178 patients with ANP were collected from our database, and the patients were divided into training (*n* = 125) and validation (*n* = 53) cohorts. Through computed tomography image acquisition, the volume of interest segmentation, and feature extraction and selection, a pure radiomics model in terms of POF prediction was established. Then, a clinic-radiomics model integrating the pure radiomics model and clinical risk factors was constructed. Both primary and secondary endpoints were compared between the high- and low-risk groups stratified by the clinic-radiomics model.

**Results:**

According to the 547 selected radiomics features, four models were derived from features. A clinic-radiomics model in the training and validation sets showed better predictive performance than pure radiomics and clinical models. The clinic-radiomics model was evaluated by the ratios of intervention and mechanical ventilation, intensive care unit (ICU) stays, and hospital stays. The results showed that the high-risk group had significantly higher intervention rates, ICU stays, and hospital stays than the low-risk group, with the confidence interval of 90% (*p* < 0.1 for all).

**Conclusions:**

This clinic-radiomics model is a useful instrument for clinicians to evaluate the incidence of POF, facilitating patients' and their families' understanding of the ANP prognosis.

## 1. Introduction

Accumulating evidence suggests that the incidence of acute pancreatitis caused by various reasons (e.g., biliary lithiasis and hypertriglyceridemia) continues to increase worldwide [1, 2]. Although the majority of cases are self-limited, up to 20% of cases can progress to necrosis of the pancreatic or peripancreatic tissue, and requiring advanced medical and intensive care [3, 4]. The acute necrotizing pancreatitis (ANP) mortality rate is gradually decreasing in recent years, as the treatment pattern has shifted from aggressive surgical debridement to minimally invasive approaches. However, severe acute pancreatitis remains the most intractable digestive disease [5, 6]. In clinical settings, organ failure (OF) is demonstrated to be the leading cause of mortality in patients with ANP. More than 45% of patients with OF present prolonged intensive care unit (ICU) admission and poor prognosis [7, 8].

Over the past decade, there have been improvements in understanding the natural history and causes of OF in ANP [9]. Nevertheless, few studies have identified the risk factors for predicting the occurrence of OF in patients with ANP. As a dynamic process, OF is dominated by systemic inflammatory response syndrome (SIRS) and sterile injury during the first 2 weeks and/or secondary to infection during weeks 2–4 [10–12]. It has been reported that persistent SIRS could predict the development of OF, although the specificity of the prediction is unsatisfactory [13]. Hence, it has been of long interest to establish a scoring system to predict the incidence of persistent organ failure (POF) during the course of ANP.

Radiomics refers to the extraction and analysis of a large number of quantitative features from medical images to address clinical needs [14, 15]. As a noninvasive approach, radiomics has been widely employed for diagnosis [16, 17]. Nevertheless, the role of radiomics in the treatment of acute pancreatitis has not been fully understood. Given pancreatitis is located deeply in the retroperitoneal space and is composed of heterogeneous cells with various distributions of intensities in the images, radiomics has the potential to capture tiny lesions of pancreatitis [18]. Although normal and/or necrotic tissues of pancreatitis can reflect the characteristics of ANP, the combination of radiomics features that are extracted from necrotic and normal regions has a better capability of comprehensively presenting the severity of pancreatitis.

Therefore, the aim of this study is to establish an instrument that combines the radiomics features of contrast-enhanced computed tomography (CECT) and clinical elements (APACHE II score and persistent SIRS) to predict the incidence of POF in ANP. This will not only help physicians better estimate the severity of ANP but also optimize treatment planning for patients predicted to have POF.

## 2. Methods

### 2.1. Data Collection

This study was approved by the Ethics Committee of Sir Run Run Shaw Hospital, School of Medicine, Zhejiang University, China (Ethics No. 20200701-33) and conducted according to the Declaration of Helsinki. The clinical and imaging data of patients meeting the inclusion and exclusion criteria were collected retrospectively from our database. The inclusion criteria were as follows: (1) the patients with CECT were routinely performed within 7 days after symptoms onset and (2) the patients were diagnosed as ANP by CECT. The exclusion criteria were as follows: (1) the patients had previously received intervention treatment in other hospitals; (2) flare-up of chronic pancreatitis; and (3) there were data missing.

**Figure 1:**
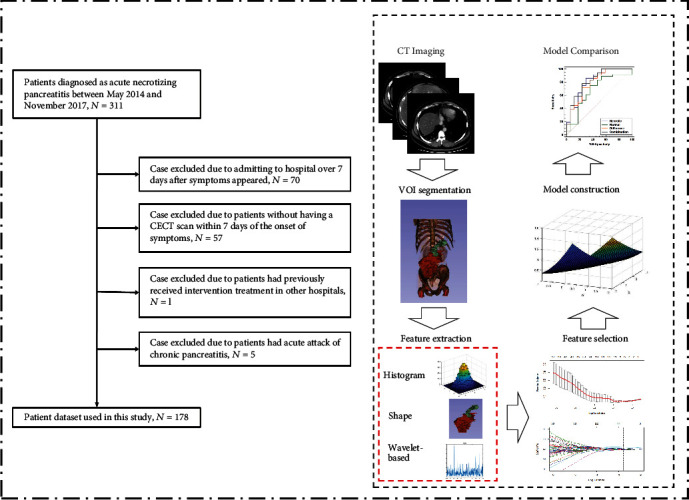
Flowchart of this study. Flowchart of patient enrollment.

The eligibility of patients is summarized in Figure 1. The enrolled patients (*n* = 178) were divided into training (*n* = 125) and validation (*n* = 53) cohorts. The demographics and laboratory investigations, including sex, age, body mass index (BMI), etiology, past history of pancreatitis, American Society of Anesthesiologists (ASA) grade, Acute Physiology and Chronic Health Evaluation (APACHE) II score, and persistent SIRS (≥48 hours), were recorded. In the case of a transferred patient, data were retrieved from the primary hospital.

### 2.2. Definition of End Points

According to the revised Atlanta classification criterion, OF was defined as the failure of the respiratory, cardiovascular, or renal system [19]. The primary end point was defined by the incidence of POF during hospitalization. Transient OF and POF were defined as OF lasting less than or over 48 hours, respectively. The predefined secondary end points included the incidences of intervention (i.e., percutaneous catheter drainage, video-assisted retroperitoneal debridement, or open necrosectomy), presence of infected necrosis (i.e., presence of gas in the necrotic collection on CECT, or positive culture of necrotic tissue obtained preprocedurally or during intervention), mechanical ventilation, ICU admission, longer hospital stay (i.e., over 30 days), and mortality.

### 2.3. Computed Tomography Image Acquisition

Sixty-four spiral contrast-enhanced CT (Somatom Definition AS, Siemens Healthineers) and Sixty-four spiral contrast-enhanced CT (LightSpeed VCT, General Electric) were used for data acquisition. Abdominal CECT scans were performed with the patient in the supine position, and the scanning ranged from the diaphragmatic domes to the ischium. After the pre-contrast scan, the patient received intravenous non-ionic iodinated contrast agent iohexol (320 mgI/mL) at a dose of 1.5 mL/kg as a bolus at a rate of 3.0 ml/s and then was injected with 50 ml normal saline. Arterial and portal venous phase images were obtained 30 and 60 seconds after injection of contrast agent, respectively. Portal venous phase images were used for evaluation because the displayed necrosis in these images was clearer. Standard Digital Imaging and Communications in Medicine (DICOM) format was used for images in this study.

### 2.4. Volume of Interest Segmentation and Feature Extraction

The regions of normal and necrotizing pancreatic tissues were contoured by an experienced radiologist and validated by another experienced radiologist independently using the ITK-SNAP software (http://www.itksnap.org/pmwiki/pmwiki.php). These two radiologists were experts in the study of diagnosed pancreatic diseases. To normalize different image specifications due to the utilization of various CT scanners, all slices were resampled to a 1 × 1 × 1 mm^3^ voxel space size, and the gray level was normalized to 64 levels for radiomics feature calculation. In this study, 547 radiomics features were extracted by MATLAB 2018b (MathWorks, Natick, MA, USA) software, including 7 shape features, 7 histogram features, 22 gray-level co-occurrence matrix (GLCM) features, 13 gray-level run-length matrix (GLRLM) features, 13 gray-level size zone matrix (GLSZM) features, 5 neighborhood gray-tone difference matrix features, and 480 wavelet-based features. The detailed full names and abbreviations of these radiomics features were presented in Supplementary Table 1. The MATLAB toolkit archive could be found at https://github.com/mvallieres/radiomics.

### 2.5. Radiomics Feature Selection and Model Construction

Considering the different scales among features, radiomics features were normalized using the Z-score method. The mean and standard deviation of the features in the training cohort were utilized to normalize the corresponding features in the validation cohort. With the low-dimensional patient sample size and high-dimensional feature size, a two-step feature selection method was used. In the training cohort (*n* = 125), each significant feature calculated by the Wilcoxon test (*p* < 0.05) was maintained. Afterward, the least absolute shrinkage and selection operator (LASSO) with five-fold cross-validation feature selection algorithm was used to select the features most robust and relevant to the status of POF, and the selected features had minimal redundancy among each other [20]. The binomial deviance criterion in the logistic regression model fitting method was used to select the best value of *λ*, which is the penalty for each variable coefficient. The *λ* with the least binomial deviance was chosen for the LASSO regression. These selected features multiplied with their coefficients calculated by LASSO were applied to construct the final radiomics signature [21].

### 2.6. Establishment and Utility of the Clinic-Radiomics Nomogram

Considering the potential effectiveness of clinical factors, a multivariable regression model integrating clinical features and the radiomics score was performed. The backward search method with the Akaike information criterion (AIC) score was employed to select the optimal combination [22]. Furthermore, the model with the lowest AIC score was employed as the optimal combined radiomics classifier to identify the status of POF in patients with ANP.

The performance of model was assessed in terms of its discrimination, calibration, and clinical utility. The receiver operating characteristic (ROC) curve was used to evaluate the prediction performance of the developed radiomics model. The validation cohort was used to verify the performance of each classifier. The model's calibration was assessed by employing calibration curves accompanied by the Hosmer–Lemeshow test [23]. The discrimination capability of the model was evaluated by the AUC and compared by the DeLong test [20]. In addition, decision curve analysis was applied to measure the clinical utility of the model.

According to the highest Youden index, the cutoff value was calculated and stratified the patients into high-risk and low-risk groups. By constructing the nomogram prediction model, we could attain a nomogram score of each patient. When the nomogram score of a patient was higher than cutoff value, the patient was stratified into the high-risk group. When the nomogram score of a patient was lower than cutoff value, the patient was stratified into the low-risk group. The predefined primary and secondary end points were compared among high-risk and low-risk groups subsequently.

### 2.7. Standard Treatment Procedure of ANP

The treatment protocol was determined by multidisciplinary team (Center of Severe Pancreatitis, Sir Run Run Shaw Hospital, Zhejiang University, China), including gastroenterologists, hepatobiliary surgeons, interventional radiologists, anesthesiologists, critical care medicine physicians, infectious disease specialists, and nutritionists. According to the evidence-based guidelines on the management of ANP, percutaneous catheter drainage into the peripancreatic space, retroperitoneum, and pelvis will not only facilitate drainage of these dependent areas but also act as an entry portal for subsequent video-assisted retroperitoneal debridement. If failed, open necrotomy was performed. Conservative medical supports were as follows: goal-directed fluid strategies based on clinical parameters; antibiotics only for patients with suspected or confirmed infection; mechanical ventilation or vasoactive drug support, if necessary; and enteral nutrition (oral or enteral tube), which was preferred rather than parenteral nutrition.

### 2.8. Statistical Analysis

Continuous variables were expressed as medians with ranges, while categorical data were presented as numbers with proportions. The Mann–Whitney *U* test was used to compare continuous variables, and the Pearson chi-square test or Fisher's test was used to compare categorical data as appropriate. LASSO feature selection was performed using the “glmnet” package. All statistical analyses were performed with R software (Version 3.4.1, www.R-project.org).

## 3. Results

### 3.1. Clinical Characteristics

From May 2014 to July 2019, 2273 patients with acute pancreatitis from our medical database were initially evaluated. Out of all studied subjects, 1013 patients who did not receive a CECT within 1 week after symptom onset were omitted, along with 1067 other patients who were not diagnosed with ANP. More subjected were further excluded, which included one patient who received intervention in a local hospital, seven patients who had a flare-up of chronic pancreatitis, and seven patients who had missing data. A total of 178 patients with ANP were eventually analyzed. The characteristics of the patients in the training (from May 2014 to May 2018) and validation (from June 2018 to July 2019) cohorts were comparable (Table 1]. 75 (60%) and 36 (67.9%) patients presented the POF in the development and validation cohorts, respectively.

### 3.2. Radiomics Feature Selection and Radiomics Model Construction

**Figure 2:**
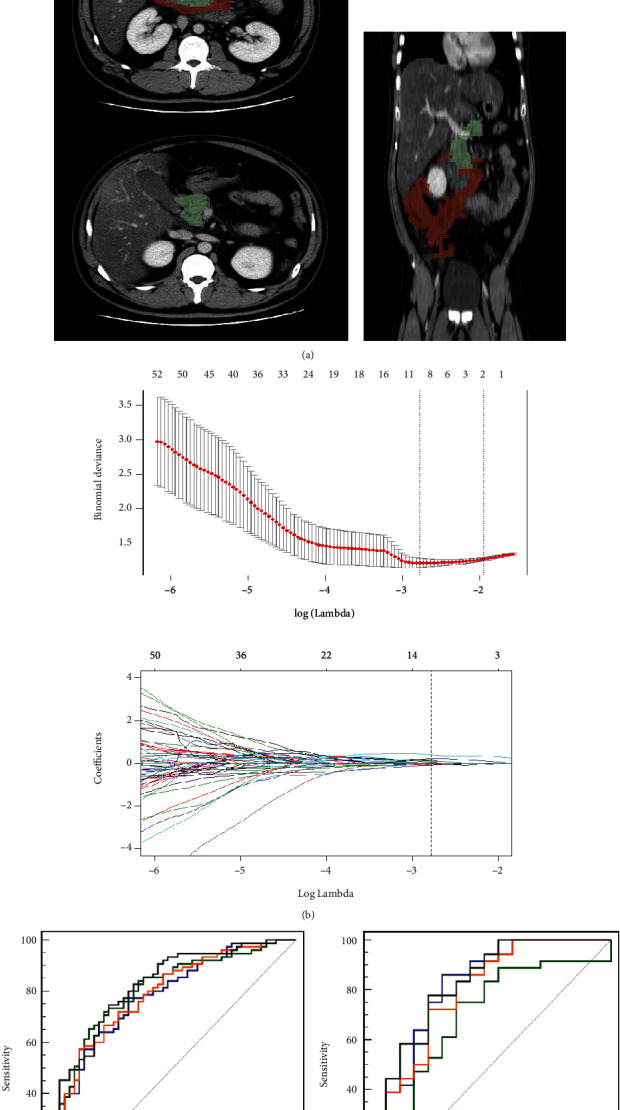
The process of pure radiomics model construction. (a) The delineation of VOI, green region is necrotizing region and red region is normal region. (b) The process of feature selection using LASSO method. The ROC curves of four radiomics models in development cohort (c) and validation cohort (d).

Among the 547 features of each type, several features showed a significant difference between the POF and non-POF groups in the training set (*n* = 125), corresponding to necrotic regions, normal regions, and values of the difference (Figure 2(a)]. The three and nine radiomics features selected by LASSO method were identified to construct models based on necrotic regions and normal regions, respectively. Moreover, the signature developed by a total of nine features (involving four necrotic features, four normal features, and one difference feature) in the LASSO algorithm showed the best prediction performance. These selected features contained VolumeML_necrotic, inf2h_necrotic, Busyness_LLL_necrotic, SVR_necrotic, Contr_normal, GLV_normal, SZE_LLL_normal, HGZE_LLL_normal, and cprom_difference. The feature of VolumeML_necotic showed the best performance in discriminating the POF status of patients with an AUC of 0.766 (0.693–0.838). Details of the procedure for feature selection were depicted in Figure 2(b). The quantitative values to represent the radiomics signature were as follows: Radiomics signature = 0.4715 + 0.2040 × VolumeML_necrotic − 0.0263 × inf2h_necrotic + 0.2039 × Busyness_LLL_necrotic − 0.0994 × SVR_necrotic + 0.1235 × Contr_normal + 0.0881 × GLV_normal − 0.0576 × SZE_LLL_normal + 0.0366 × HGZE_LLL_normal − 0.0566 × cprom_difference. The detailed description of selected radiomics features was listed in Supplementary Table 2.

### 3.3. Establishment and Comparison of Radiomics Models

Radiomics models showed promising results in predicting the status of POF in patients with ANP. For the first three models developed by features of the normal region, necrotic region, and difference between two regions, the AUC values were 0.808 (0.728–0.873), 0.776 (0.692–0.845), and 0.783 (0.700–0.851) in the training cohorts, and 0.681 (0.539–0.803), 0.817 (0.687–0.910), and 0.794 (0.661–0.893) in the validation cohorts, respectively. On this basis, the combination model (the fourth model) integrating all 1641 features showed the highest AUC values, with AUC values of 0.815 (0.736–0.879) in the training set and 0.824 (0.694–0.915) in the validation set (Figures 2(c) and 2(d)]. Additionally, the accuracies of the normal, necrotic, difference, and combination models were 74.4%, 73.6%, 69.6%, and 76% in the development cohort and 64.2%, 60.4%, 56.7%, and 64.2% in the validation cohort, respectively (Supplement Figure 1]. Therefore, the combination model was regarded as the optimal radiomics model (subsequently referred to as radiomics model).

### 3.4. The Clinic-Radiomics Nomogram

**Figure 3:**
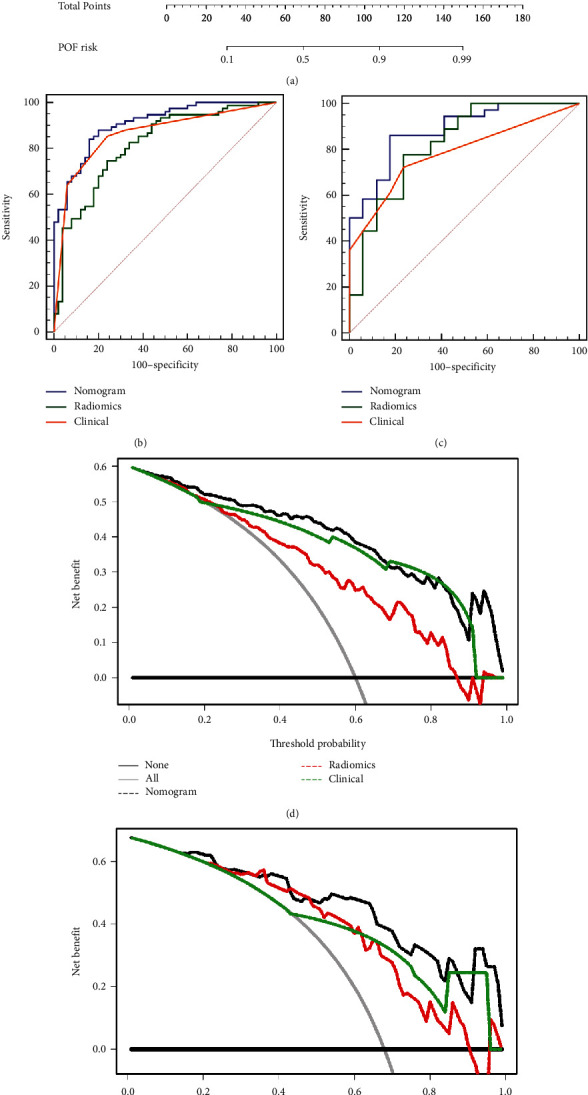
The clinic-radiomics model construction and evaluation. (a) The clinic-radiomics, combined radiomics score, SIRS stage, and APACHEII stage. The ROC curves of nomogram, radiomics, and clinical models in the development cohort (b) and validation cohort (c). Blue line represents the nomogram, green line represents the radiomics model, and yellow line represents clinical model. The decision curves of the nomogram, the radiomics, and clinical models in the development cohort (d) and the validation cohort (e). Black line represents the nomogram model, red line represents the radiomics model, and green line represents the clinical model.

The APACHE II score (≥8) and persistent SIRS presented significant differences between the POF and non-POF datasets according to the AIC score. The clinical model integrating these two factors showed promising performance, with AUCs of 0.862 (0.799–0.925) and 0.783 (0.673–0.894) in the training and validation sets, respectively. To improve the performance, logistic regression analysis incorporating the radiomics signature and clinical features was performed in the training cohort. A clinic-radiomics model (nomogram) consisting of the APACHE II score (≥8), the persistent SIRS, and the combination model (radiomics score) indicated the best capacity for predicting the status of POF (Figure 3(a)]. The calibration curve of which presented high accuracy in the training and validation cohorts (Supplement Figure 2A and Supplement Figure 2B].

The prediction accuracy of the high-risk group reached 84.8% and 73.6% in the development and validation cohorts, respectively. The scatter plots for nomogram scores were shown in Supplement Figure 2C and Supplement Figure 2D. The AUC values of the clinic-radiomics model were 0.907 (0.842–0.952) and 0.882 (0.764–0.955) in the development and validation sets, respectively. Compared with the pure radiomics and clinical models, the ROC curves of the nomogram showed promising higher AUC values in both the training and validation cohorts (Figures 3(b) and 3(c)]. The significant improvement was observed between clinic-radiomics model and clinical model by Delong test. The specific calculated *p*-values among all constructed models were recorded in Supplementary Table 3. Moreover, the decision curve analysis (DCA) also showed that the clinic-radiomics model had better clinical utility than the two other models (Figures 3(d) and 3(e)].

### 3.5. End Points in Low-Risk and High-Risk Patients

To further validate the reliability of the clinic-radiomics model, the primary and secondary end points were compared between the low-risk and high-risk groups based on the clinic-radiomics nomogram (Table 2]. When the nomogram score of patient was higher than 69.1, patient was stratified into the high-risk group, otherwise, the patient was stratified into the low-risk group. The incidence of POF in the high-risk group was obviously higher than that in the low-risk group (both *p* < 0.001 in the two cohorts). On the other hand, other factors like the rates of intervention, mechanical ventilation, ICU admission, and longer hospital stays were increased in the high-risk group (*p*-value <0.1 for all factors).

## 4. Discussion

In this study, we developed and validated a novel scoring system constructed by integrating radiomics clinical features to effectively predict the incidence of POF in patients with ANP. As suggested by a previous study indicating that the median interval between the onset of OF and death was only 3 days, there is great significance to predicting the incidence of OF, especially the incidence of POF, and improving the efficiency of comprehensive treatment in patients with ANP [23, 24]. Generally, OF is an important determinant of outcome in patients with ANP. The characteristics of OF, including severity, specific type (e.g., respiratory, cardiac, and renal), number of organs affected, and persistent time of OF, have also been demonstrated to affect the clinical course and outcome [8, 9, 13]. Patients with early fulminant POF have been considered to have higher mortality than those developing POF later. Hence, we established a clinic-radiomics model to noninvasively and quantitatively predict the incidence of POF in patients with ANP.

To our knowledge, this is the first study to provide radiomics feature insight into POF in patients with ANP, although a handful of scoring systems have been developed to judge the severity and prognosis of ANP or severe acute pancreatitis. In terms of radiomics, the combination model achieved the most favorable performance by incorporating features in necrotic regions, normal regions, and the difference values. Then, the optimal clinic-radiomics model was established and showed satisfactory accuracy and generalization. Of note, two advantages of this clinic-radiomics model should be acknowledged.

Initially, the large-scale characteristics of CECT scans were analyzed to extract histogram-, shape-, and texture-based features from the region of interest in patients with ANP [24]. To overcome the time consumption, the few features captured, and observer specificity, radiomics through artificial intelligence (AI) patterns could automatically extract meaningful information from CECT scans [25, 26]. In this study, several features selected by the LASSO algorithm were identified as crucial elements for POF in patients with ANP, which provides additional value beyond simple radiological variables seen by the naked eye [27]. The feature of total volume showed the best performance by using univariate analysis, and this result was consistent with that of a previous study [28]. The difference in contrast features between necrotic tissue and normal tissue also played an important role in stratifying the status of POF. The reason for this result may be that the status of POF is strongly correlated with the variation in pancreatic tissue in each patient. We also found that wavelet-based features made great contributions to model construction. These features were extracted from images decomposed by undecimated 3D wavelet transforms. Therefore, wavelet-based features can provide multiscale information on pancreatic tissue. Given that traditional radiomics predictive models for the prognosis of various cancers are limited to the tumor region, the optimal model was established by comparing the radiomics characteristics from normal and necrotic regions on CECT scans. By analyzing the underlying pathophysiology on medical images, this radiomics model comprehensively predicted the incidence of POF in ANP [29].

In addition, clinical indicators such as persistent SIRS and the APACHE II score (≥8) are closely related to the incidence of POF in patients with ANP. Pathologically, the uncontrolled activation of inflammation causes injury to pancreatic tissues and progresses to necrosis [30–33]. Several inflammatory parameters (e.g., interleukin-6, C-reactive protein, and procalcitonin) induce the incidence of POF as mediators of the acute-phase response. The early phase caused by persistent SIRS is proposed as the early peak of POF during the development of severe acute pancreatitis [30, 31]. As the APACHE II score represents the chronic health state individually, high scores indicate a damaged compensatory ability for stress [32, 33]. In line with our previous study that showed that SIRS and the APACHE II score are connected with the severity and prognosis of hypertriglyceridemia-induced acute pancreatitis, both are proposed as risk factors for the incidence of POF in patients with ANP [34]. Therefore, the step-up approach (percutaneous drainage, minimally invasive debridement, and open necrotomy) can be adopted to eliminate detrimental metabolites and endogenous toxins, decreasing the incidence of POF by minimizing SIRS and improving the patients' general condition.

There were several study limitations that should be addressed. Since this clinic-radiomics model was derived from a single institution with a limited number of patients, an external validation trial should be warranted. Additionally, our method included ANP patients with different etiologies, and the generalization would be restrictive to some extent. Given the limited number of cases, different kinds of POF (namely respiratory, cardiovascular, or renal failures) have not been distinguished. Hence, more evidence is still needed to study whether the clinic-radiomics model would be justified in different settings.

In conclusion, this clinic-radiomics model shows a remarkable capability for predicting POF in patients with ANP. It could be a useful instrument for clinicians to evaluate the incidence of POF and facilitate patients' and their families' understanding of the prognosis of ANP. This could prevent subsequent disputes and lead to compliance in patients with unfavorable outcomes. Moreover, optimized treatment planning, such as early intervention, might be considered for high-risk patients according to the clinic-radiomics model.

## Figures and Tables

**Table 1:** Clinical characteristics.

	Development cohort (*n* = 125)	Validation cohort (*n* = 53)	*p*-value	
Age, years			0.629
Median	46	48	
Range	17–84	24–79	
Gender			0.358
Male	84 (67.2)	40 (75.5)	
Female	41 (32.8)	13 (24.5)	
BMI, kg/m^2^			0.583
≥25.0	73 (58.4)	34 (64.2)	
<25.0	52 (41.6)	19 (35.8)	
ASA grade			0.065
≥III	37 (29.6)	24 (45.3)	
<III	88 (70.4)	29 (54.7)	
Etiology			0.335
Hyperlipidemia	47 (37.6)	24 (45.3)	
Biliary	56 (44.8)	24 (45.3)	
Others	22 (17.6)	5 (9.4)	
Past history of AP			0.223
Present	34 (27.2)	20 (37.7)	
Absent	91 (72.8)	33 (62.3)	
APACHE II, points			0.203
≥8	57 (45.6)	18 (33.9)	
< 8	68 (54.4)	35 (66.1)	
Persistent SIRS			0.130
Present	76 (60.8)	25 (47.2)	
Absent	49 (39.2)	28 (52.8)	
Persistent OF			0.407
Present	75 (60.0)	36 (67.9)	
Absent	50 (40.0)	17 (32.1)	
Intervention			0.721
Present	42 (33.6)	20 (37.7)	
Absent	83 (66.4)	33 (62.3)	
Infected necrosis			0.656
Present	32 (25.6)	16 (30.2)	
Absent	93 (74.4)	37 (69.8)	
Mechanical ventilation			0.951
Present	40 (32.0)	16 (30.2)	
Absent	85 (68.0)	37 (69.8)	
ICU admission			0.347
Present	84 (67.2)	31 (58.5)	
Absent	41 (32.8)	22 (41.5)	
Hospital stay (over 30 days)			0.721
Present	42 (33.6)	20 (37.7)	
Absent	83 (66.4)	33 (62.3)	
Mortality			0.815
Present	10 (8.0)	3 (5.7)	
Absent	115 (92.0)	50 (94.3)	

Data are presented as median with range or number with percentage. BMI, body mass index; ASA, American Society of Anesthesiologists; AP, acute pancreatitis; APACHE, Acute Physiology and Chronic Health Evaluation; SIRS, systemic inflammatory response syndrome; OF, organ failure; ICU, intensive care unit.

**Table 2:** Second end points between high-risk and low-risk groups based on nomogram model.

	Training cohort (n =125)		*p* value	Validation cohort (n =53)		*p* value
	High-risk (*n* = 76)	Low-risk (*n* = 49)		igh-risk (*n* = 26)	Low-risk (*n* = 27)	
Intervention			0.001			0.001
Present	34	8		17	3	
Absent	42	41		9	24	
Infected necrosis			0.090			0.054
Present	24	8		13	3	
Absent	52	41		13	24	
Mechanical ventilation			<0.001			0.054
Present	37	3		13	3	
Absent	39	46		13	24	
ICU stay, days			<0.001			<0.001
	70	14		22	9	
	6	35		4	18	
Hospital stay, days			0.002			0.037
	34	8		14	6	
	42	41		12	21	
Mortality			0.777			0.973
Present	7	3		2	1	
Absent	69	46		24	26	

Data are presented as median with range or number with percentage. ICU, intensive care unit.

## Data Availability

The data that support the findings of this study are available from the corresponding author upon reasonable request.

## Supplementary Materials

Supplementary Materials Supplement Table 1 The radiomics features names and its abbreviations in this study. Supplement Table 2 The description of selected radiomics features. Supplement Table 3 The *p*-values of comparison in different radiomics models.

Supplementary Materials Supplementary Figure 1 The scatter plots between different radiomics model. The scatter plots of normal radiomics model in development cohort (A) and validation cohort (B). The scatter plot of necrotizing radiomics model in development cohort (C) and validation cohort (D). The scatter plot of difference radiomics model in development cohort (E) and validation cohort (F). The scatter plot of combined radiomics model in development cohort (G) and validation cohort (H). Supplementary Figure 2 The calibration curves and scatter plots of combined nomogram model. The calibration curves of the combination nomogram in the development cohort (A) and validation cohort (B). The scatter plots of the nomogram scores in the development model (C) and validation model (D) were developed by combined features. The blue points indicated patients with non-POF, and the red points indicated patients with POF.
