# Supplementary material for: A Clinic-Radiomics Model for Predicting the Incidence of Persistent Organ Failure in Patients with Acute Necrotizing Pancreatitis
Source: Gastroenterol Res Pract. 2023 Aug 17;2023:2831024. doi: 10.1155/2023/2831024 (PMC10449595; doi:10.1155/2023/2831024)
Supplement: Supplementary Materials — Supplement Table 1 The radiomics features names and its abbreviations in this study. Supplement Table 2 The description of selected radiomics features. Supplement Table 3 The p-values of comparison in different radiomics models. [file 2831024.f1.docx]

| **Supplement Table 1 The radiomics features names and its abbreviations in this study** | | | | | |
| --- | --- | --- | --- | --- | --- |
| **Histogram** | **GLCM** | **GLSZM** | **GLRLM** | **NGTDM** | **Shape** |
| Variance | Autocorrelation(autoc) | Small Zone Emphasis(SZE) | Short Run Emphasis(SRE) | Coarseness | Surface Volume Ratio(SVR) |
| Skewness | Contrast(contr) | Large Zone Emphasis(LZE) | Long Run Emphasis(LRE) | Contrast | Compactness1(Cpt1) |
| Kurtosis | Correlation(corr1) | Gray-Level Non-uniformity(GLN) | Gray-Level Non-uniformity(GLN.1) | Busyness | Compactness2(Cpt2) |
| Mean | Correlation2(corr2) | Zone-Size Non-uniformity(ZSN) | Run-Length Non-uniformity(RLN) | Complexity | Spherical Disproportion(SphDisp) |
| Energy | Cluster Prominence(cprom) | Zone Percentage(ZP) | Run Percentage(RP) | Strength | Sphericity |
| Entropy | Cluster Shade(cshad) | Large Zone Low Gray-Level Emphasis(LZLGE) | Low Gray-Level Run Emphasis(LGRE) |  | Volume maximum level(VolumeML) |
| Uniformity | Dissimilarity(dissi) | Large Zone High Gray-Level Emphasis(LZHGE) | High Gray-Level Run Emphasis(HGRE) |  | Surface Area(SA) |
|  | Energy(dissi) | Small Zone Low Gray-Level Emphasis(SZLGE) | Short Run Low Gray-Level Emphasis(SRLGE) |  |  |
|  | Entropy(entro) | Small Zone High Gray-Level Emphasis(SZHGE) | Short Run High Gray-Level Emphasis(SRHGE) |  |  |
|  | Homogeneity(homom) | Low Gray-Level Zone Emphasis(LGZE) | Long Run Low Gray-Level Emphasis(LRLGE) |  |  |
|  | Homogeneity2(homop) | High Gray-Level Zone Emphasis(HGZE) | Long Run High Gray-Level Emphasis(LRHGE) |  |  |
|  | Maximum probability(maxpr) | Gray-Level Variance(GLV) | Gray-Level Variance(GLV.1) |  |  |
|  | Sum average(savgh) | Zone-Size Variance(ZSV) | Run-Length Variance(RLV) |  |  |
|  | Sum of squares Variance(sosvh) |  |  |  |  |
|  | Sum variance(svarh) |  |  |  |  |
|  | Sum entropy(senth) |  |  |  |  |
|  | Difference variance(dvarh) |  |  |  |  |
|  | Difference entropy(denth) |  |  |  |  |
|  | Information measure of correlation1(inf1h) |  |  |  |  |
|  | Information measure of correlation2 (inf2h) |  |  |  |  |
|  | Inverse difference normalized (indnc) |  |  |  |  |
|  | Inverse difference moment normalized (idmc) |  |  |  |  |
| GLCM, Gray-level co-occurrence matrix; GLRLM, Gray-level run-length matrix; GLSZM, gray-level size zone matrix; NGTDM, neighbourhood gray-tone difference matrix. | | | | | |

**Supplement Table 2 The description of selected radiomics features.**

| Feature Name | Description |
| --- | --- |
| VolumeML | The volume of ROI. |
| inf2h | The assessment of correlation between the probability distributions of i and j (quantifying the complexity of the texture). |
| Busyness_LLL | The busyness metric is used to gauge the extent of variation between a pixel and its immediate surroundings. LLL indicates features gained by performing low-pass wavelet filter on all 3 axes. |
| SVR | The ratio of surface area to volume |
| Contr | Contrast is a measure of the local intensity variation, favoring values away from the diagonal (i=j). |
| GLV | GLV measures the variance in gray level intensities for the zones. |
| SZE_LLL | A quantifiable metric used to determine the distribution of smaller size zones in a given material. LLL indicates features gained by performing low-pass wavelet filter on all 3 axes. |
| HGZE_LLL | HGLZE is a measure used to determine the distribution of higher gray-level values in an image. LLL indicates features gained by performing low-pass wavelet filter on all 3 axes. |
| Cprom | A metric that quantifies the extent of skewness and asymmetry present in GLCM (Gray Level Co-occurrence Matrix) |

| **Supplement Table 3 The p-values of comparision in different radiomics model.** | | |
| --- | --- | --- |
| Models | Training cohort (n=125) | Validation cohort (n=53) |
| Combination *vs* Necrotic | 0.010 | 0.782 |
| Combination *vs* Normal | 0.862 | 0.069 |
| Combination *vs* Difference | 0.007 | 0.097 |
| Combination *vs* Clinical | 0.319 | 0.630 |
| Clinic-radiomics *vs* Clinical | 0.020 | 0.021 |
| Clinic-radiomics *vs* Radiomics | 0.004 | 0.202 |
| The Combination model is the Clinic-radiomics model | | |
