# Supplementary material for: A Clinic-Radiomics Model for Predicting the Incidence of Persistent Organ Failure in Patients with Acute Necrotizing Pancreatitis
Source: Gastroenterol Res Pract. 2023 Aug 17;2023:2831024. doi: 10.1155/2023/2831024 (PMC10449595; doi:10.1155/2023/2831024)
Supplement: Supplementary Materials — Supplementary Figure 1 The scatter plots between different radiomics model. The scatter plots of normal radiomics model in development cohort (A) and validation cohort (B). The scatter plot of necrotizing radiomics model in development cohort (C) and validation cohort (D). The scatter plot of difference radiomics model in development cohort (E) and validation cohort (F). The scatter plot of combined radiomics model in development cohort (G) and validation cohort (H). Supplementary Figure 2 The calibration curves and scatter plots of combined nomogram model. The calibration curves of the combination nomogram in the development cohort (A) and validation cohort (B). The scatter plots of the nomogram scores in the development model (C) and validation model (D) were developed by combined features. The blue points indicated patients with non-POF, and the red points indicated patients with POF. [file 2831024.f2.docx]

**Supplementary Figure 1 The scatter plots between different radiomics model.** The scatter plots of normal radiomics model in development cohort (A) and validation cohort (B). The scatter plot of necrotizing radiomics model in development cohort (C) and validation cohort (D). The scatter plot of difference radiomics model in development cohort (E) and validation cohort (F). The scatter plot of combined radiomics model in development cohort (G) and validation cohort (H).

**
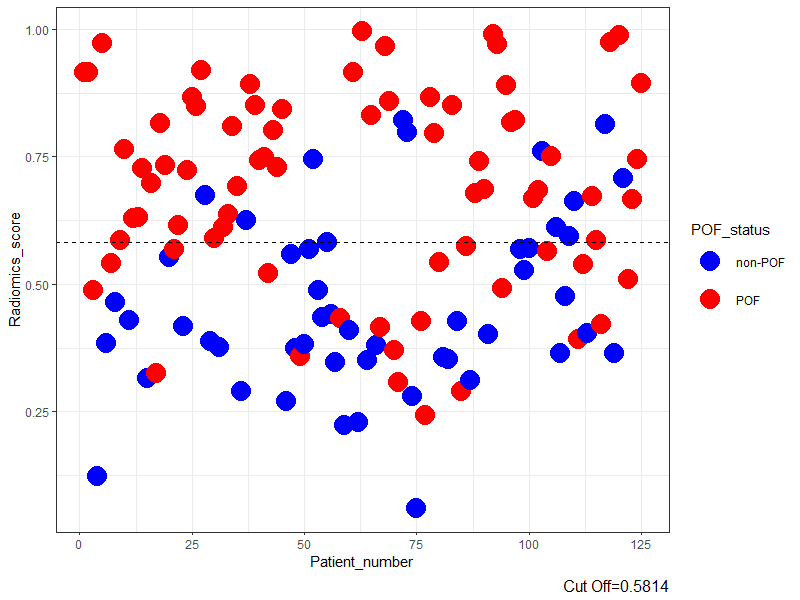

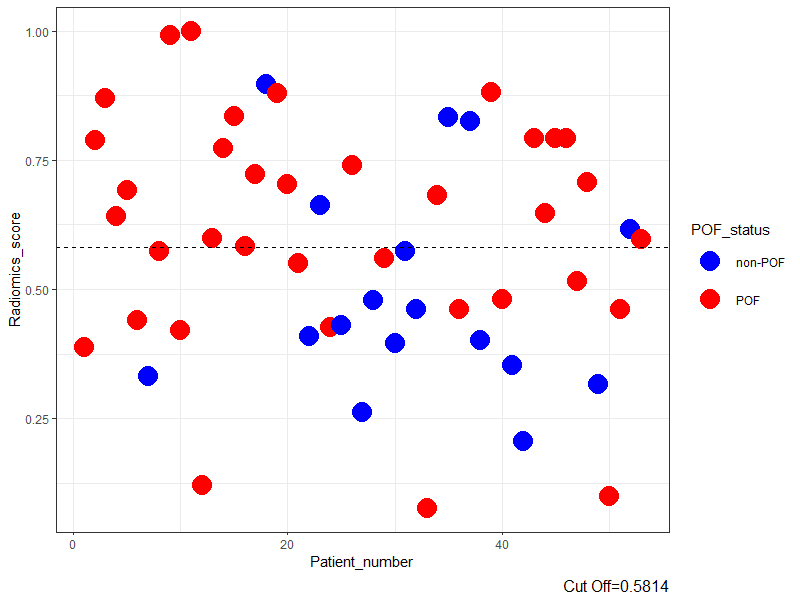

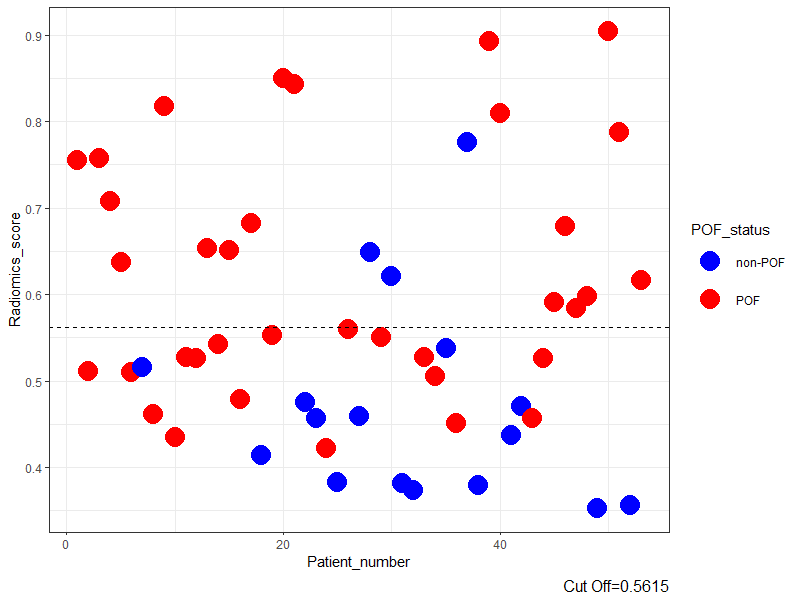

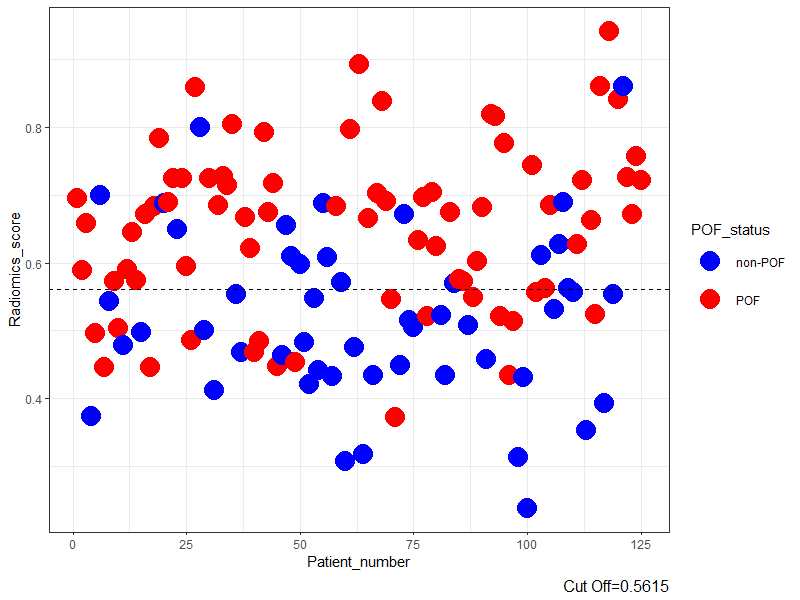

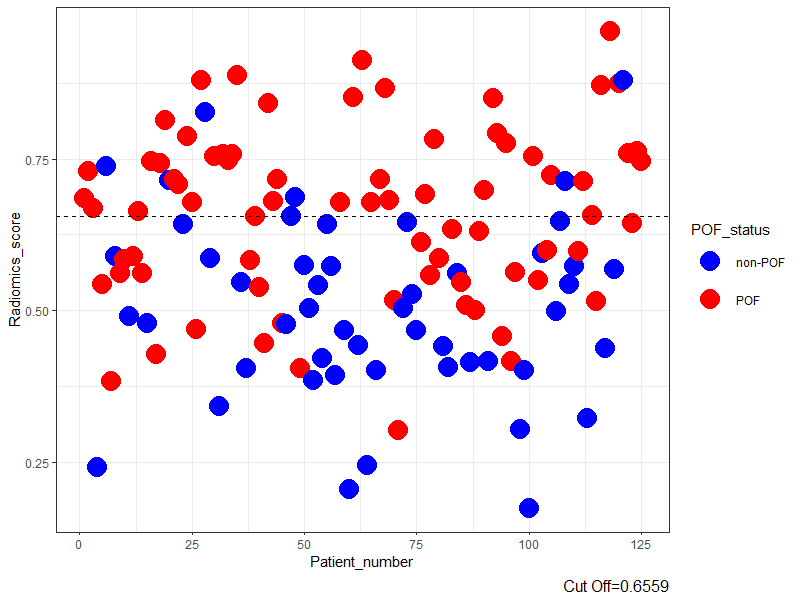

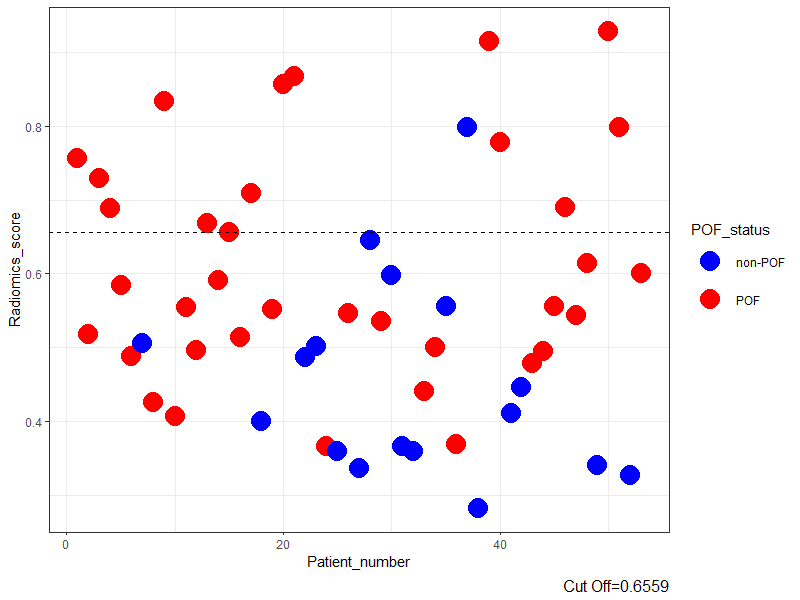

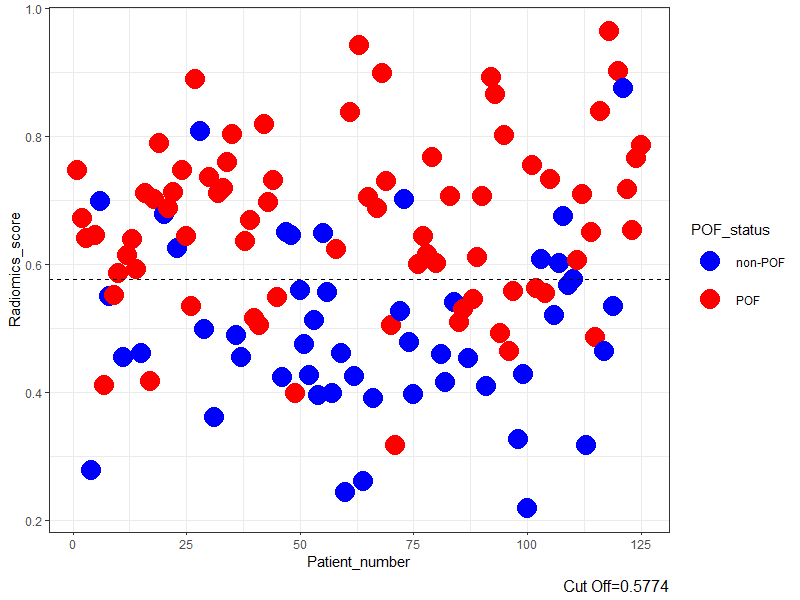

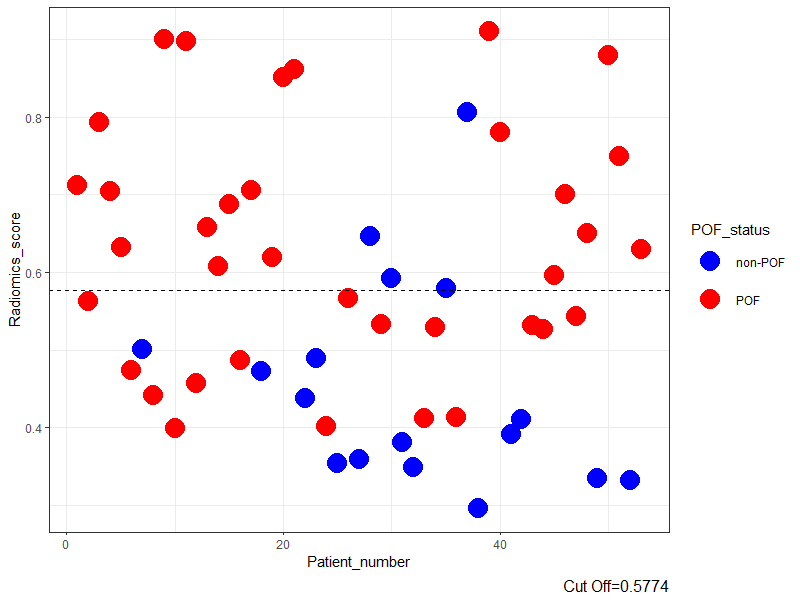
**

**A**

**B**

**C**

**D**

**E**

**F**

**G**

**H**

**Supplementary Figure 2 The calibration curves and scatter plots of combined nomogram model.** The calibration curves of the combination nomogram in the development cohort (A) and validation cohort (B). The scatter plots of the nomogram scores in the development model (C) and validation model (D) developed by combined features. The blue points indicated patients with non-POF and the red points indicates patients with POF


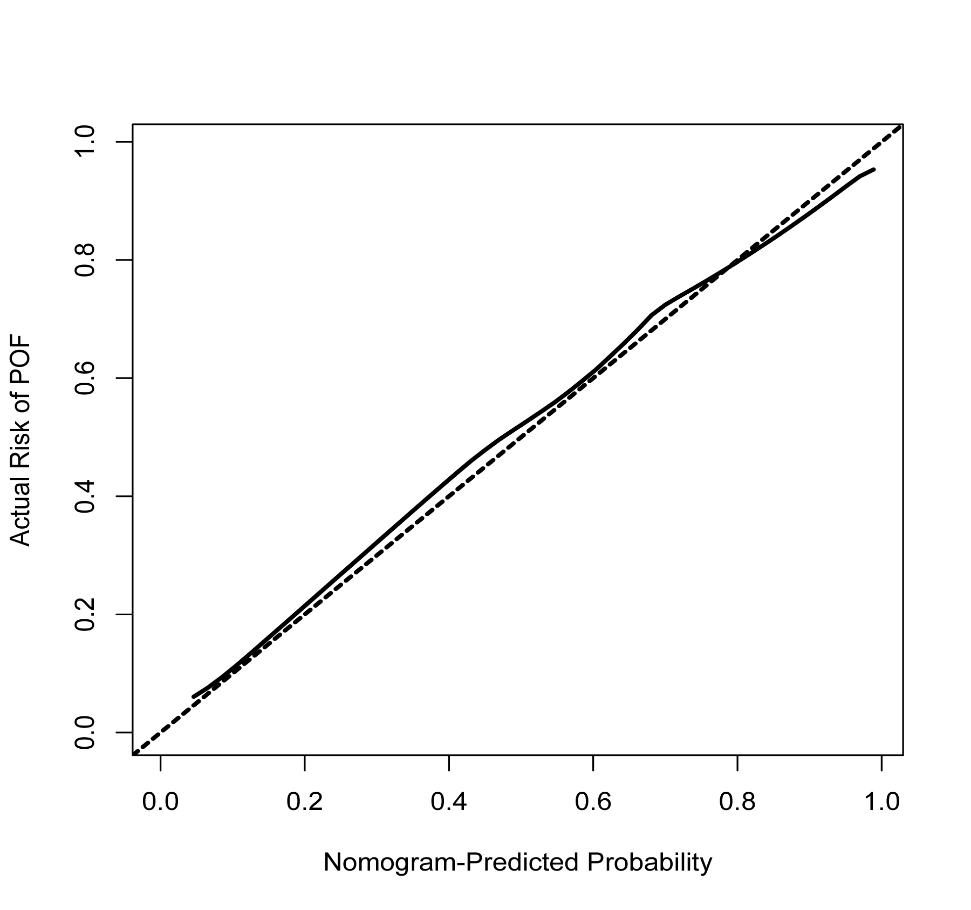

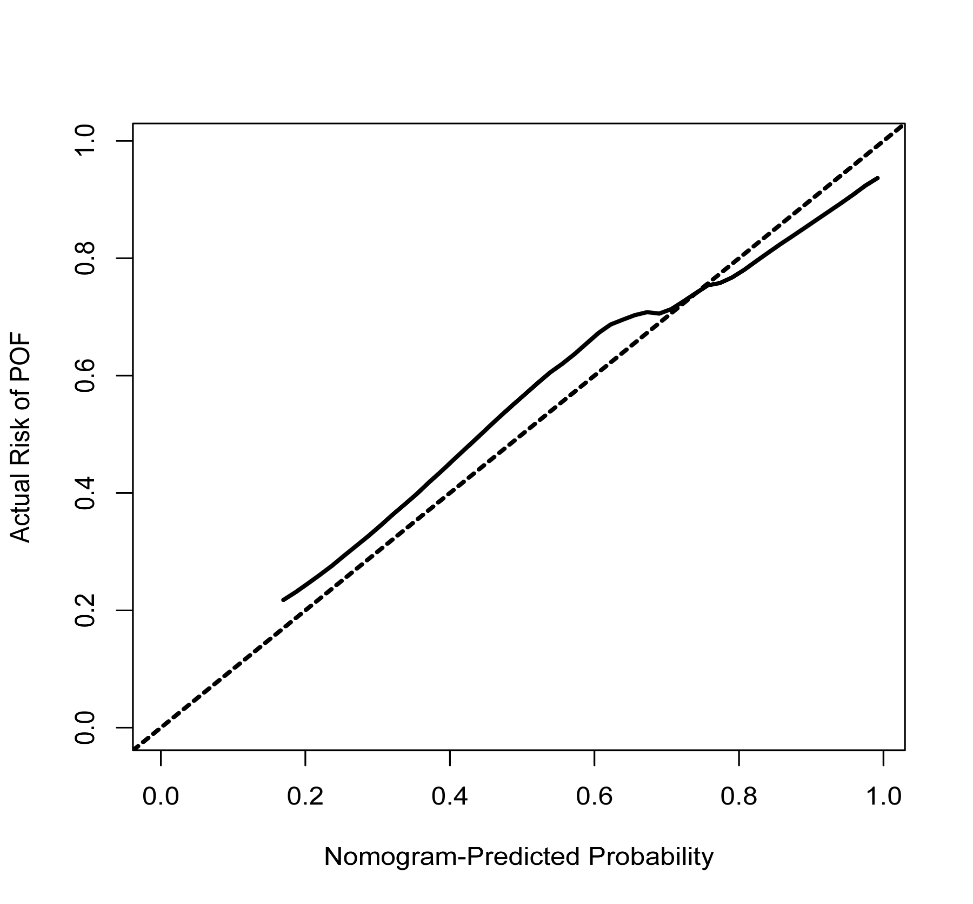

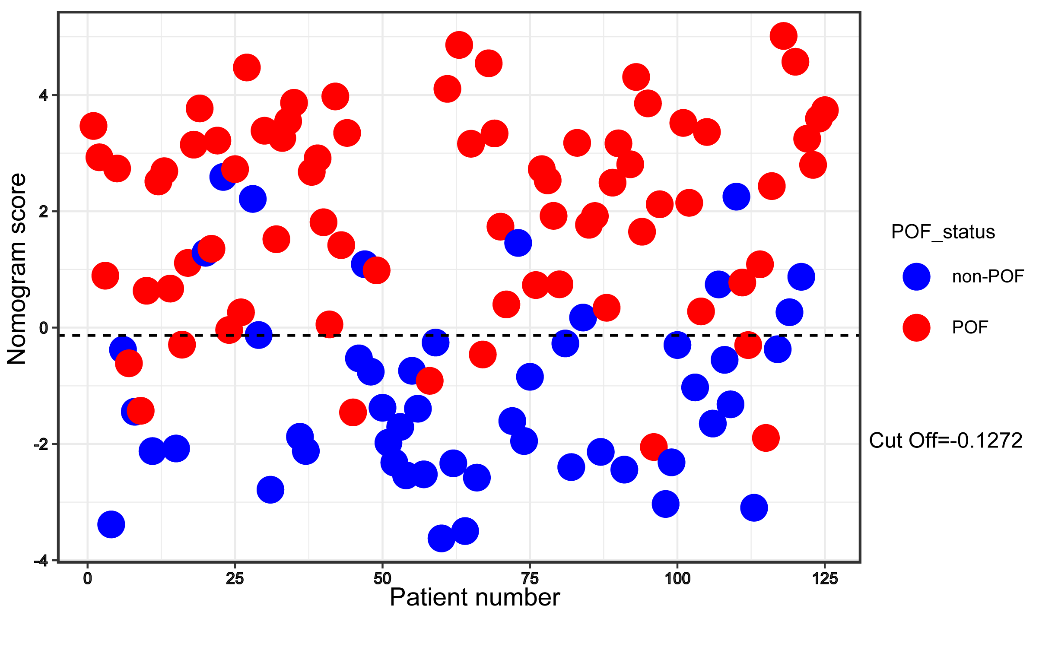

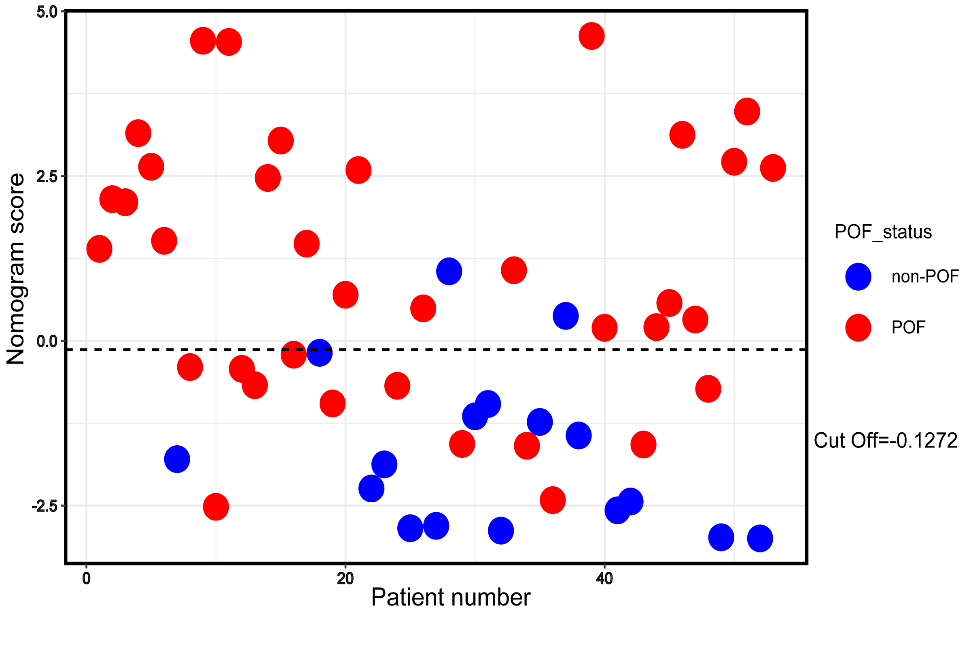


**A**

**B**

**C**

**D**
